# Durability Properties of Concrete Supplemented with Recycled CRT Glass as Cementitious Material

**DOI:** 10.3390/ma14164421

**Published:** 2021-08-06

**Authors:** Dušan Zoran Grdić, Gordana Aleksandar Topličić-Ćurčić, Zoran Jure Grdić, Nenad Srboljub Ristić

**Affiliations:** Faculty of Civil Engineering and Architecture, University of Niš, 18000 Niš, Serbia; gordana.toplicic.curcic@gaf.ni.ac.rs (G.A.T.-Ć.); zoran.grdic@gaf.ni.ac.rs (Z.J.G.); nenad.ristic@gaf.ni.ac.rs (N.S.R.)

**Keywords:** cathode ray tube glass, strength, durability, freeze-thaw resistance, de-icing salts, sulphate attack, alkali-silica reaction

## Abstract

This paper presents the testing of the durability of concrete where a part of cement was replaced with ground panel cathode ray tube glass (CRT) finer than 63 µm. The percentage of cement replaced with glass is 5%, 10%, 15%, 20%, and 35%, by mass. The highest percent share of mineral admixtures in CEM II (Portland-composiste cement) cement was chosen as the top limit of replacement of cement with glass. In terms of the concrete durability, the following tests are performed: freeze-thaw resistance, freeze-thaw resistance with de-icing salts-scaling, resistance to wear according to the Böhme test, sulfate attack resistance, and resistance to penetration of water under pressure. A compressive strength test is performed, and shrinkage of concrete is monitored. In order to determine the microstructure of concrete, SEM (Scanning Electron Microscopy) and EDS (Energy Dispersive X-ray Spectroscopy) analyses were performed. The obtained research results indicate that the replacement of a part of cement with finely ground CRT glass up to 15% by mass has a positive effect on the compressive strength of concrete in terms of its increase without compromising the durability of concrete. The results obtained by experimental testing unequivocally show that concrete mixtures made with partial replacement (up to 15%) of cement with finely ground CRT glass have the same freeze-thaw resistance, resistance to freeze/thaw with de-icing salt, resistance to wear by abrasion, and resistance to sulfate attack as the reference concrete. In terms of environmental protection, the use of CRT glass as a component for making concrete is also very significant.

## 1. Introduction

It is well known that different waste materials are very successfully implemented in the contemporary construction industry. To date, extensive research on fly ash, blast furnace slag, and silica dust use, as well as on mineral admixtures when making concrete and concrete prefabricates, has been conducted. The potential to use waste glass, including cathode ray tube (CRT) glass, for making of new products or as an admixture to the existing ones is being intensively investigated. This kind of research intensified particularly in the period after CRT TV sets and computer monitors were replaced in the market by the advanced technology of thin film transistor (TFT) and liquid crystal display (LCD) screens. Cathode ray tube glass represents a considerable part of electronic waste (e-waste). E-waste globally increases at a far higher rate than other solid waste materials. According to the data of the United Nations University, in 2018 solely, a total of 49.8 million tons of e-waste was generated in the world, CRT comprising a considerable part of it [1]. It is estimated that yearly around 50–150 thousand tons of phased-out CRT screens end up in landfills [2]. The European waste catalog classified CRT glass as hazardous waste because of the presence of lead [3]. In that sense, it is not possible to use it for the production of new glass containers without previous removal of hazardous lead.

One possibility to use waste glass is to replace a part of the natural aggregate for concrete making [4,5,6,7,8,9,10]. A problem that may arise on this occasion is the potential emergence of the alkali-silicate reaction (ASR). Recycled glass contains a high percentage of amorphous silicon which can react with alkali from the cement whereby the ASR gel is created [11], which in the presence of humidity in a longer time period has a tendency to expand and can lead to the destruction of concrete. Experimental research proved that the size of a grain of ground glass has a considerable effect on ASR [12,13,14]. Maraghechi et al. [12] concluded that the size of cracks inside the glass grains created during the process of crushing and grinding could be correlated to ASR.

The other option is the use of finely ground waste glass as a substitution for part of the cement, supplementary cementitious material (SCM), when making concrete. Such use of waste glass would have both economic and environmental benefits considering that cement-producing industries emit almost 7% of the total amount of global CO_2_ emissions. Previous research determined that grinding the glass with a high content of amorphous silicon into a fine dust (particles finer than 100 µm) activates its pozzolanic reactivity, which is the basic precondition for its use as a supplementary cementitious material [14,15,16,17,18,19,20,21,22]. Nassar et al. [23] determined a considerable increase in the compressive strength of the concrete in relation to the reference concrete at the age of 56 days, which was ascribed to the pozzolanic activity of ground mixed-color milled waste glass and formation of a denser concrete microstructure. In the research conducted by Aliabdo et al. [24], it was determined that by adding 15% of ground waste building demolition glass to the entire amount of cement, the increase in the compressive strength of concrete is on average 16% compared to the reference concrete. Furthermore, tensile strength, absorption voids ratio, and density are improved as a result of using 10.0% glass powder cement replacement. Sadiqul et al. [25] determined that the optimum level of replacement of cement with ground clear and colored glass is 20%, whereby the compressive strength of mortar and concrete are slightly increased (2%), while the cement production cost is reduced by 14%. Several years of research by Omran et al. [26] produced a conclusion that the addition of finely ground bottle glass of mixed colors has a positive effect on the increase in splitting tensile strength, flexural strength, and modulus of elasticity which is the result of the pozzolanic reaction of glass and improved concrete microstructure.

In the research of the use of CRT glass as SCM, special attention is paid to the durability of such cement composites. Papers [23,27,28,29,30,31,32] present research results of individual properties of concrete, which were also the subject matter of the research in this paper, and they refer to the durability of concrete. Related to this, Nassar et al. [23] concluded that the use of milled waste glass as partial replacement of cement in recycled aggregate concrete results in enhanced durability characteristics such as sorption, chloride permeability, and freeze–thaw resistance through the improvement in pore system characteristics. Related to this, Nassar et al. [23] concluded that the use of milled waste glass as a partial replacement for cement in recycled aggregate concrete results in enhanced durability characteristics such as sorption, chloride permeability, and freeze–thaw resistance through the improvement in pore system characteristics. Kim et al. [27] concluded that the ground glass sludge, a byproduct of glass plate manufacture, improves the resistance of concrete to freezing, freezing and thawing, and de-icing salt and chloride ion penetration. Zidol et al. [28] and Lee et al. [29] also determined that the presence of finely ground mixed soda-lime glass and glass sludge in concrete dramatically reduces chloride ion penetration, regardless of the water/binder ratio. It was concluded that the presence of glass improves the concrete microstructure, which directly causes the reduction in concrete porosity and water absorption. The conclusion of the Ling and Poon [30] research is that the shrinkage due to concrete drying is reducing with the increase in CRT glass share in the cement composite. Such an effect of waste glass on the concrete shrinkage can be explained by the fact that glass does not absorb water, whereby water is only needed to dampen the glass particles [31,32].

Based on the review of a large number of papers, only some of which have been mentioned in the introduction, it can be concluded that only a small number of researches on the durability of concrete were performed with finely ground CRT glass. One of the goals set for this research is proving the potential use of CRT recycled glass as a partial replacement for cement in concretes, which are expected to have the same durability properties as the reference concrete. Moreover, the goal of this experimental research is to determine the ultimate percentage of replacement of cement with finely ground CRT glass, where the durability properties of concrete are at such a level to allow their practical use. In order to achieve the set goals, we conducted very extensive and comprehensive research that lasted several years. In this paper, we presented the research results referring only to the durability of concrete, and for that purpose solely, 350 concrete test specimens were made. The basic motivation for conducting this study is solving the problem of CRT glass disposal, which is a severe environmental problem that can, to a great extent, be solved by proving it can be used as a component of concrete. The importance of the study is reflected in the successfully proven potential for the use of CRT as a replacement for cement while even making certain economic gains. Namely, the practice up to date in Serbia is that the collected CRT glass is exported to other countries and the countries taking in the recycled CRT glass are paid for that.

The experimental research presented in this paper is directed towards testing the durability of concrete where a part of cement is replaced by finely ground panel CRT glass. In terms of concrete durability, the following tests are conducted: freeze-thaw resistance, freeze-thaw resistance with de-icing salts-scaling, resistance to wear according to the Böhme test, sulfate attack resistance, and resistance to penetration of water under pressure. Additionally, the compressive strength is measured and the shrinkage of concrete is monitored. For the purpose of observing the concrete microstructure, SEM and EDS analyses are performed.

## 2. Materials and Methods

### 2.1. Materials

#### 2.1.1. Cementitious Materials

**Figure 1:**
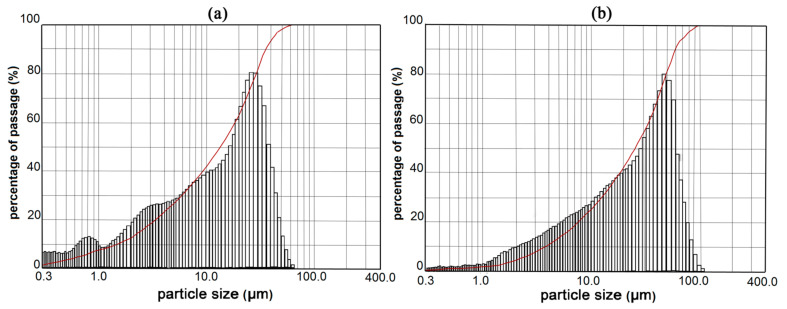
Particle size distribution of Portland cement CEM I 52.5R (**a**) and CRT glass (**b**).

Portland cement CEM I 52.5R (CRH—Novi Popovac, Serbia) is used for making the concrete mixtures, according to standard EN 197-1. Panel CRT glass is taken from the recycling center of Niš, Serbia. Large shards of CRT glass are ground using the laboratory ball mill so that the glass particles are finer than 0.063 mm. The physico-chemical composition of finely ground CRT glass and cement is presented in Table 1. The chemical composition of CRT glass is provided so that it can be compared with the chemical composition of the cement used in the experimental testing in order to point out its potential pozzolanic properties. On the other hand, it is well known that glass contains several hazardous substances such as chromium, manganese, cobalt, nickel, cadmium, mercury, lead, etc. In order to determine the impact of concrete with the addition of CRT glass on the environment, a leaching test has been performed according to the standard EN 1744-3. The obtained results of the chemical analysis of eluates indicate that the content of potentially hazardous metals is far below the permissible limit values. Regarding that, the paper discusses the durability of concrete; the results of the leaching test are not presented here. Ground glass has a specific mass of 2.84 g/cm^3^ and the Blaine specific area of 245 m^2^/kg, which is around 40% less than the cement. The particle size distribution of cement and ground CRT glass is presented in Figure 1. More than 63% of glass grains are finer than 36 μm, while 42% are finer than 20 μm. Around 25% of the particles of cathode ray tube glass are finer than 10 μm. Considering there is no standard for CRT glass that defines the testing of the activity index, the standard EN 450-1 t.5.3.2. which defines this for fly ash is used. In addition to the replacement of cement with finely ground CRT glass in the amount of 25% (in accordance with the standard EN 450-1), the activity index testing is conducted on mortars where the percentage of replacement amounts are 5%, 10%, 15%, 20%, and 35%. These percentages of replacement of cement with CRT glass are later used for making concrete. The results of the activity index tests are presented in Table 2. When up to 15% of the cement is replaced with CRT glass, the criteria for the activity index at 28 and 90 days are met. When up to 20% of the cement is replaced with CRT glass, the activity index is not reached at the age of 90 days for 3%, the same happens for 25% of cement replaced at the same age, and for 4%. In the case of 35% of cement replaced, conditions for the activity index are not satisfied. Irrespective of the activity index obtained results, in the following experimental research, all the mentioned percentages of replacement of cement with glass are used.

#### 2.1.2. Aggregate

**Figure 2:**
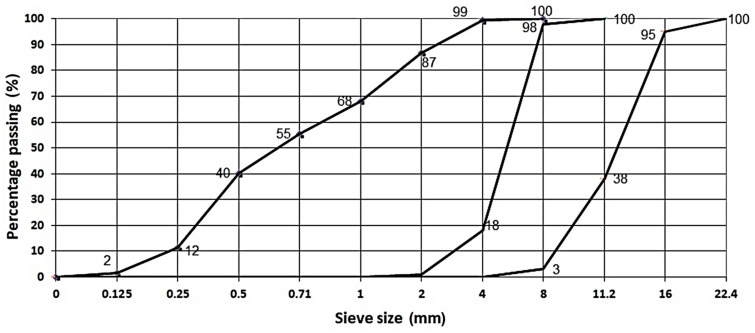
Particle size distribution of fractions 0/4 mm, 4/8 mm, 8/16 mm of river aggregate.

In the experiment three fractions of river aggregate are used (0/4 mm, 4/8 mm, and 8/16 mm). The particle size distribution of aggregate fractions is determined using the dry sieving method according to EN 933-1 and shown in Figure 2.

### 2.2. Mix Design

In order to test the impact of the replacement of a part of cement with finely ground CRT glass on the properties of fresh and hardened concrete, six concrete mixtures are made, i.e., a total of 350 specimens for experimental testing. The target slump class is S3 (*slump 100–130 mm*) of fresh concrete, tested according to the standard EN 12350-2. The shares of individual fractions of aggregate in the mixture are 43% (*fraction 0/4 mm*), 23% (*fraction 4/8 mm*), and 34% (*fraction 8/16 mm*). Reference concrete is made with 400 kg of cement and 1800 kg of three-fraction aggregate. The water/binder ratio (w/b) is constant and amounts to 0.438. Superplasticizer on the basis of polycarboxylate is used for making the concrete mixes, which reduces the amount of water. The percentage of cement replaced with CRT glass amounts to 5%, 10%, 15%, 20%, and 35%, compared to the mass of cement. The highest percent of the share of mineral admixtures in CEM II cement was chosen as the top limit of replacement of cement with glass. The markings of concrete mixtures are made corresponding to the percentage of cement replacement (Table 3), where WG is an abbreviation for waste glass. The complete composition of concrete mixtures is presented in Table 3.

### 2.3. Test Methods

#### 2.3.1. Testing of Fresh Concrete

Concrete consistency testing is performed according to the provisions of the standard EN 12350-2 [33], density according to EN 12350-6 [34], and content of the air in the concrete according to the standard EN 12350-7, pressure gauge method [35].

#### 2.3.2. Testing of Hardened Concrete

In Table 4, there is a list of conducted tests of hardened concrete and standards they followed. Further, the text provides additional explanations of testing procedures in cases when they are performed according to national standards.

##### Alkali-Silicate Reactivity (ASR)

Reference mortar (E) is made with fine river aggregate and cement, with no addition of glass. In mortar WG1, 35% of cement is replaced by CRT glass having fineness 0.063/0.090 mm, while in mortar series WG2, 35% of cement is replaced by the ground CRT glass finer than 0.063 mm. The goal of the research is to check the alkali-silicate reactivity of CRT glass in a mortar and to investigate the effect of the fineness of glass on the intensity of ASR. Such a percentage of cement replaced with finely ground CRT glass is chosen because it is later found to be the highest percentage of cement replaced in concrete. The other reason is the assumption that the potential ASR will be more manifest if the replacement of cement is 35% in comparison to the lower percentages of replacement.

##### Freeze-Thaw Resistance of Concrete

Prior to the test, nine specimens of concrete in the shape of a cube having a side of 150 mm, 28 days old, are immersed in water having a temperature of +20 ± 3 °C until saturated. After that, they are exposed to 200 cycles of subsequent freezing and thawing. One cycle consists of freezing the specimens at the temperature of −20 ± 2 °C for 4 h and thawing in the water having temperature +20 ± 3 °C for 4 h. According to the standard SRPS U.M1.206 [38], concrete is resistant to freezing if the average compressive strength of concrete samples exposed to freezing is no less than 75% of the average strength of the specimens which are not exposed to freezing at an equivalent age. The equivalent age of concrete cubes (Te), which are cured in water only, is calculated for a cycle of freezing for 24 h according to the formula:

(1) $$T_{e}=a+0.8\cdot n\;\;\;\;\;\;\;\;\;$$

where:

a—is the age of samples in a day at the start of the test;

n—is the number of cycles of alternating freezing and thawing while testing the compressive strength.

The compressive strength of the frozen specimens is tested after the completed 200th cycle, and the reduction in the compressive strength of concrete is calculated in comparison to the reference specimens cured in water to the equivalent age according to the formula:

(2) $$\Delta f_{p,200}=\frac{f_{E}-f_{S}}{f_{E}}\cdot 100\left(\%\right)$$

where:

$f_{E}$—is the compressive strength of reference specimens cured in water to the equivalent age in MPa;

$f_{S}$—is the compressive strength of specimens after 200 cycles of freezing in MPa;

$\Delta f_{p,200}$_—_the reduction in compressive strength of concrete after 200 cycles of freezing in %.

##### Freeze-Thaw Resistance with de-Icing Salts-Scaling

Freeze-thaw resistance with de-icing salts-scaling is estimated by measuring the mass of the material scaled from the surface of the samples after 28 cycles of freezing and thawing. After 7, 14, and 28 cycles, visual inspection of samples is performed, scaled material is collected, its quantity measured, and replacement of the 3% solution of NaCl is performed.

##### Testing of Shrinkage in the Air

Concrete shrinkage testing is performed according to the standard UNI 6555 [42] on concrete prisms having dimensions 100 × 100 × 500 mm. Concrete is cast into the appropriate steel molds with the previously fitted reference pins. After 24 h, the specimens are demoulded and cured in water, having a temperature of 20 ± 4 °C, for the duration of 48 h. After a total of 72 h from the moment of making, the samples are taken out of water, wiped with wet flannel fabric, and the initial measuring is taken using a digital indicator. The following sample measurements are taken at the ages of concrete of 4, 7, 14, 21, 28, 60, 90, and 180 days. The variation in the specimen length ∆l_sc_ (t), caused by the shrinkage of concrete in the air, is calculated in relation to the first-initial measurement after 72 ± 0.5 h.

##### Testing of Resistance to Sulfate Attack

Each concrete series comprises 18 samples of cylinder-shaped specimens having a diameter of 100 mm and a height of 100 mm. Half of all specimens are reference specimens that are cured in saturated lime water until the moment of testing. The other half of the specimens are, at the age of 28 days, immersed and maintained in a 5% solution of Na_2_SO_4_ until the moment of testing. The entire 5% solution of Na_2_SO_4_ in the curing vessels is replaced in a way to retain the pH value of the solution. The pH value of the Na_2_SO_4_ solution is je ~7.5, i.e., ~12.5 in the case of saturated lime water. Sulfate attack resistance of concrete series with added CRT glass is determined by comparing the compressive strength of reference samples of each series with the samples exposed to the sulfate attack after the exposure of concrete to the sulfate solution in the duration of 3, 6, and 12 months. Testing of the compressive strength was conducted according to the standard ASTM C1231-14 [44]. Apart from testing the strength variation, a visual inspection of the possible damage of the samples cured in the sulfate solution is performed.

##### SEM and EDS Analyses

SEM and EDS analyses are performed in order to acquire knowledge of the concrete microstructure. All concretes are observed and analyzed, but this paper displays only the samples representing the following concrete series: reference, WG15 and WG35. The age of the samples during the analysis of the microstructure is 90 days.

## 3. Results and Discussion

### 3.1. Characteristics of Fresh Concrete with Added Finely Ground CRT Glass

**Figure 3:**
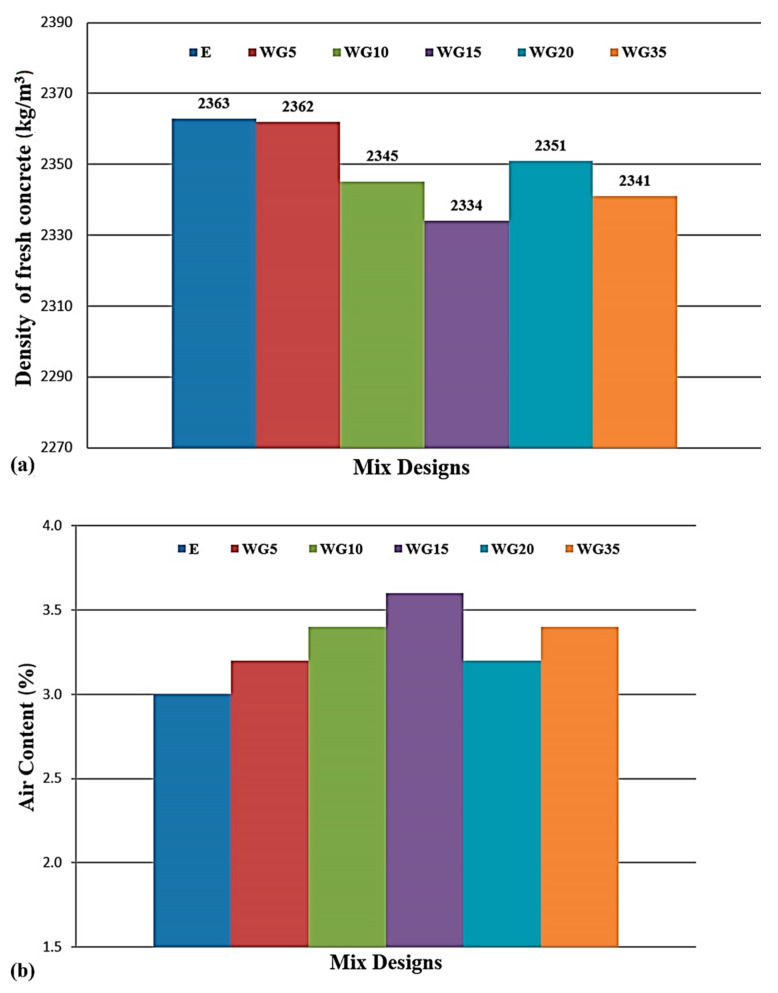
Density (**a**) and air content (**b**) of fresh concrete.

Slump test results are in the range of 100 mm to 130 mm, class S3, which has been the goal when designing concrete mixtures. The density of fresh concrete practically does not change, irrespective of the percentage of cement replaced by CRT glass (Figure 3a). For instance, in the case of the series with the highest share of glass in the mixture-WG35, the reduction in the density was 0.9%. The difference in the value of a specific gravity of cement (3.15 g/cm^3^) and finely ground CRT glass (2.84 g/cm^3^) has not had a considerable impact on the change of density of fresh concrete. This can be explained by the fact that when replacing cement with 35% of glass, it constitutes only around 5% of the concrete volume and therefore cannot have a considerable impact on the variation of density. The content of entrained air in concrete mixtures ranges between 3.0% and 3.6% (Figure 3b). Considering that the value of entrained air, even in the case of the reference series, is above the usual value of 2%, and taking into account that the air-entraining agent is not used, it is determined that the implemented superplasticizer causes the mild effect of air entrainment in the concrete mix. The experimental series of concrete where a part of cement is replaced with glass has a slightly higher percentage of entrained air than the reference concrete. The highest value of entrained air, of 3.6%, is measured in the WG15 series, which is 0.6% higher than the reference concrete. Based on the obtained results, which are very similar, it is not possible to clearly define to what extent the presence of finely ground CRT glass affects this property of fresh concrete.

### 3.2. Compressive Strength

**Figure 4:**
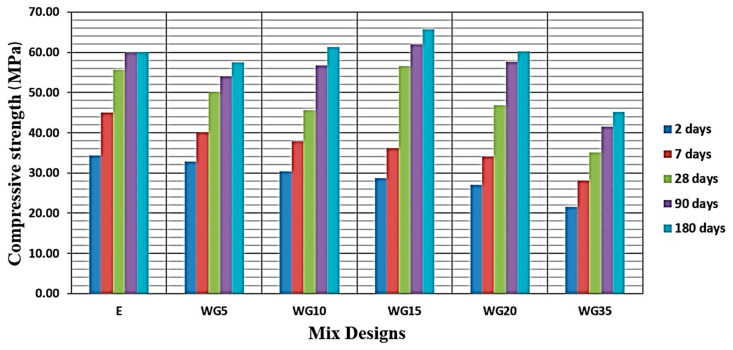
Graphic presentation of achieved compressive strengths of concrete series in time.

Testing of compressive strength is conducted on concrete cubes having the sides of 150 mm at the ages of 2, 7, 28, 90, and 180 days, and the strength at each age was the average of three specimens. As expected with the increasing age of concrete, of all series of concrete, there is an increase in compressive strength (Figure 4). At an early age of concrete of 2 and 7 days, the reference concrete has the highest values of compressive strength. For the early age period (young concrete), one can make a preliminary conclusion that with an increase in the replacement share of cement with CRT glass, the compressive strength of concrete decreases, and the higher replacement percentage means the more prominent decrease. For instance, series WG20 and WG35 at the age of 7 days have as much as 24.4% and 37.6% lower strength than the reference series. The results of compressive strength tests at the age of 28 days demand a correction of the previously made conclusions. At the age of 28 days, WG15 concrete reaches the compressive strength of the reference concrete, while other concretes continue to exhibit lower compressive strengths. At the age of 90 days, WG15 concrete has 3.6% higher compressive strength than the reference concrete, while WG10 and WG20 concretes considerably reduced the gap behind the reference concrete, and it is now only 5.2% and 3.5%, respectively. The final test at 180 days demonstrated that the highest compressive strength is demonstrated by WG15 concrete, around 9.4% higher than the reference concrete. Moreover, WG10 and WG20 fully reached the compressive strengths of the reference concrete. WG5 concrete has a 4.2% lower strength than the reference concrete, while WG35 exhibits a considerably lower strength of 24.8% (Figure 4).

The nature of the pozzolanic reaction of glass is such that it occurs later, in respect to the cement hydration process, and that is most intensive after day 28. Based on the obtained results, it has been determined that the pozzolanic reaction considerably contributes to the compressive strength at the age of 90 days as well. This can explain why the difference between the achieved values of compressive strengths of the reference concrete and concrete series where up to 20%, the cement that was replaced with glass is reducing. Such results are in accordance with the test results of other authors [23,25,28].

### 3.3. Alkali-Silicate Reactivity

**Figure 5:**
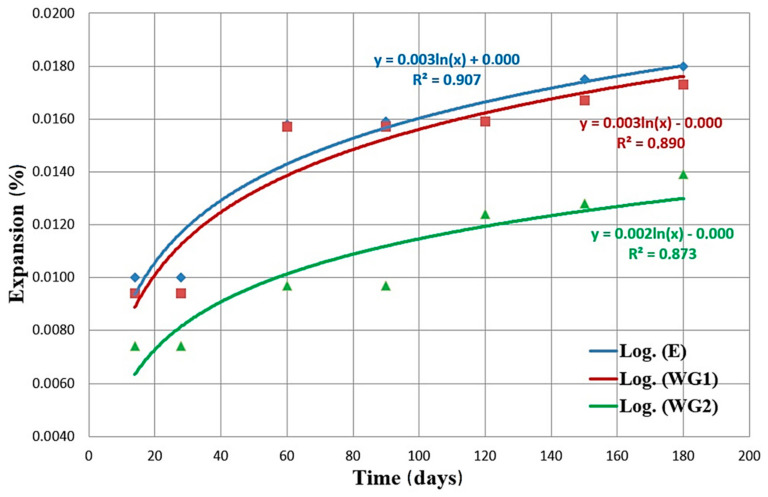
Expansion of mortar prisms with the addition of CRT glass in the function of time.

Based on the tests results presented in the diagram (Figure 5), it can be concluded that mortars WG1 and WG2 had a lower value of expansion than reference concrete at all ages. At the ages of 180 days, the values of mortar prisms E, WG1, and WG2 expansions are +0.0180%, +0.0173%, and +0.0139%, respectively.

Compliant with the provisions of ASTM C33-13 [45], expansion values after 6 months do not exceed the limit value of +0.10% in all three series of mortars. Additionally, it can be concluded that the mortar with more finely ground glass WG2 has by far the lowest value of expansion in relation to the other two mortars. At the end of the test, WG2 exhibits 22.8% lower shrinkage than WG1 and the reference mortar. Therefore, the higher fineness of glass in the case of the WG2 mortar contributes to the reduction in expansion. The review of the literature shows that the explanation for such an effect of glass on the occurrence of ASR can be found in [46]. Free SiO_2_ in finely ground glass, which is an amorphous material, will be consumed during the pozzolanic reaction, and it will react with other compounds to form a mineral phase. Dissolved silicon dioxide will be built into the crystal grid of the cement gel and will not be available for the process of the alkali-silicate reaction, which, as a rule, occurs much later than the pozzolanic reaction.

### 3.4. Freeze-Thaw Resistance of Concrete

**Figure 6:**
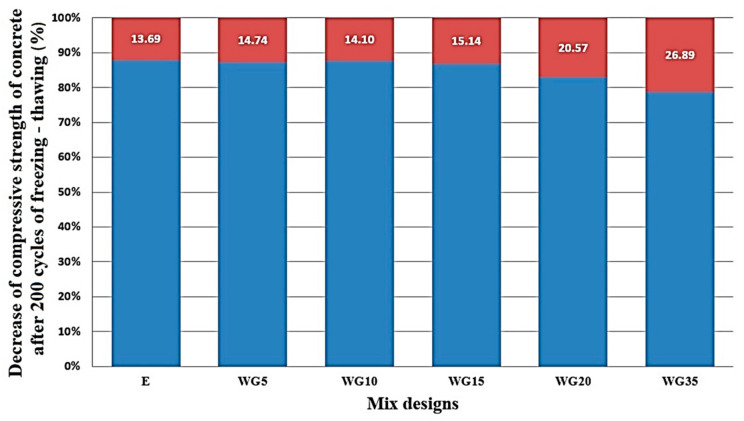
Percentage of reduction in compressive strength of concrete after 200 cycles of freezing.

The reduction in the compressive strength of concrete after 200 cycles of freezing is presented in Figure 6. In the diagram shown, the level of 100% represents the value of strength in percent of corresponding reference samples cured in water up to the equivalent age for each experimental mixture of concrete, while the percentage in the red field shows the effective decline of the strength of each series. The series where up to 15% of cement is replaced with finely ground CRT glass, after 200 cycles of freeze and thaw, exhibit a drop in strength of 14–15%, the WG20 series has a strength decrease of 20.57%, while only the WG35 series has a decline in strength higher than 25%, which means that this series can be considered non-resistant to this action. Kim et al. [27] tested the durability of concrete in terms of resistance to freezing-thawing, according to the provisions of the standard ASTM C666. Similar results were reached by Nassar et al. [23] when testing freezing-thawing resistance of concrete mixtures made with the aggregate of recycled concrete and with partial replacement of cement with ground food container glass (the replacement level was 20%, by cement mass). The authors conclude that the use of ground glass as a partial replacement of cement in concrete with recycled glass improves durability in terms of the freezing-thawing resistance, explaining that by adding ground glass to the cement matrix, the pore system becomes improved, and the sealing effect by the glass particles is exhibited and Ca(OH)_2_ is used up in the C-S-H gel due to the pozzolanic activity of glass.

### 3.5. Resistance of Concrete to Freeze/Thaw with De-Icing Salt

Table 5 shows the value of the cumulative quantity of scaled material after 28 cycles of simultaneous action of freezing/thawing and de-icing salt for each individual specimen (3 specimens for each concrete mixture), as well as the visual description of the damage. Concretes where cement is replaced up to 15% with CRT glass, are resistant to freezing/thawing in the presence of de-icing salt. Weak scaling of all three concrete samples occurs in the WG20 series. The WG35 series exhibits more considerable damage of specimens whereby individual aggregate grains are clearly visible, and this concrete does not meet the criterion prescribed for the exposure class XF2 (maximum average scaling 0.30 mg/mm^2^) in the standard SRPS U.M1.206 [38].

Kim et al. [27] tested the durability of concrete in terms of resistance to simultaneous actions of freezing-thawing and defrosting salt according to the provisions of the standard ASTM C672. The series with 5% and 10% of replacement of cement with recycled glass proved to be more resistant than the reference series in the case of this research. One of the explanations for such a result is the consequence of the formation of hydration phases during the pozzolanic reaction of glass, which caused a reduction in permeability to fluids. Hyeongi Lee et al. [29] replaced 20% of cement with two types of recycled glass. After the conducted testing of freezing-thawing action and defrosting salt, their results showed on average 30% lower mass loss than the reference concrete made with the pure PC. The literature data are in partial agreement with the test results obtained in this experiment. The explanation can be found in the following facts: tests in the papers [27,29] were conducted with different types of glass and in compliance with the American regulations, while in [27] the reference concrete was made with 20% of fly ash that replaced cement. The detailed literature review did not reveal an identical case of testing resistance of concrete with CRT glass added in terms of simultaneous action of freezing/thawing and de-icing salt.

### 3.6. Depth of Penetration of Water under Pressure

**Figure 7:**
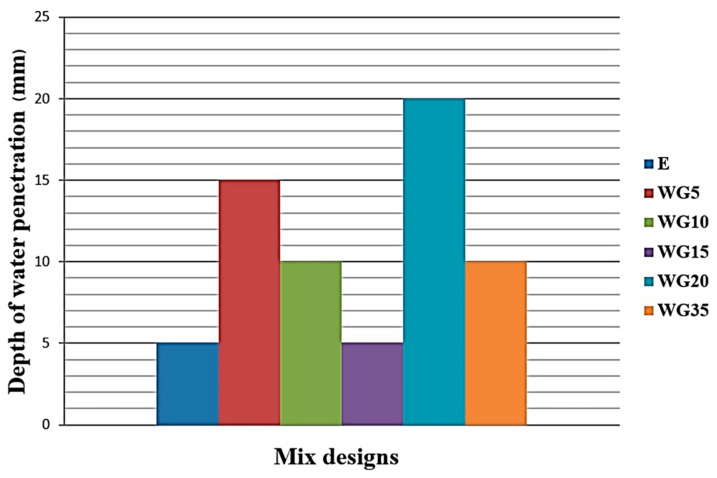
Depth of water penetration through concretes with different percentages of replacement of cement with CRT glass, rounded to the closest 5 mm.

The highest value of penetration of water under pressure is measured in WG20 concrete, but it was only 20 mm (Figure 7). The series with the highest percentage of cement replaced with WG35-glass has a very low penetration of water under pressure—as much as 10 mm, which may indicate that the finely ground glass grains had a role of a sealant in concrete. Many authors, among which Nevil [47], considered the water penetration of 30 mm the top limit of water tightness. Based on the obtained results (Figure 7), no particular impact of the addition of CRT glass on the resistance to the action of water under pressure can be determined. In general, all concrete series can be characterized as resistant to water under pressure attacks. It can be concluded that the depth of water penetration is only 5 mm to 20 mm.

### 3.7. Resistance to Wear by Abrasion

**Figure 8:**
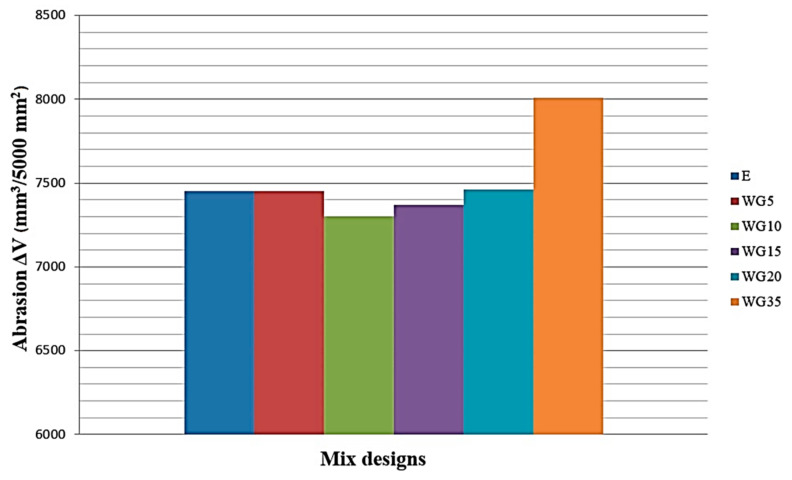
Impact of the presence of CRT glass on concrete resistance to wear by abrasion.

The effect of the presence of ground CRT glass on the resistance to wear by abrasion is shown in Figure 8. It can be noticed that in the case of the series where 20% of cement is replaced with glass, the loss of mass due to abrasion is fairly uniform. In the case of concrete with the highest percentage of replacement, WG35, there is a 7.5% reduction in resistance to abrasion in comparison to the reference concrete. The microscopy analysis of pure ground-glass (Figure 12a) reveals that grains have smooth surfaces and sharp edges. It is obvious that in this case, the quality of the transit zone between the grains of ground glass and cement matrix, and the micro-texture of the concrete surface, cause exactly such results of resistance to abrasion.

### 3.8. Testing of Shrinkage due to Drying in Air

**Figure 9:**
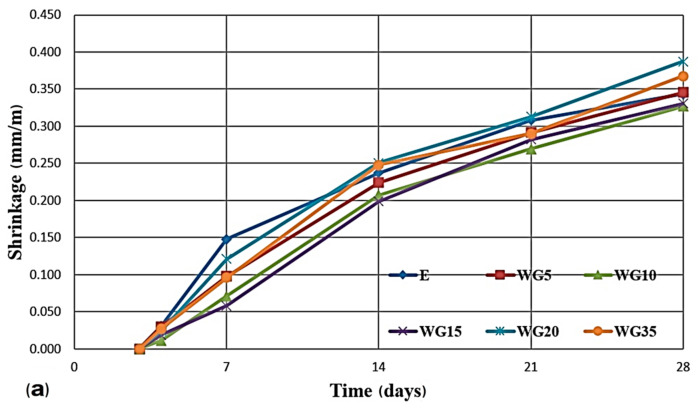

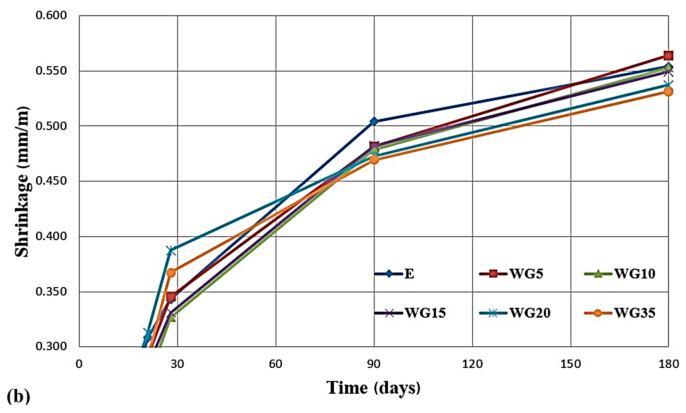
Shrinkage of concrete series in air during first 28 days (**a**) and shrinkage of concrete series in air in the period from day 28 to day 180 (**b**).

Figure 9a shows the concrete shrinkage measured during the first 28 days of testing, while Figure 9b shows the shrinkage occurring in the period from day 28 to day 180. In tests at 4, 7, 14, and 21 days of concrete mixtures having up to 20% of glass instead of cement, the shrinkage values are lower than the values of reference concrete shrinkage. If in this period, the compressive strengths of concrete mixtures with glass replacement of cement up to the mentioned level are observed, it can be concluded that those series have lower strengths than the reference concrete. From day 21 to day 180, these series exhibit a higher increase in compressive strength than the reference concrete, which, when it comes to shrinkage, corresponds to the period when the shrinkage of the mentioned series increases and approaches to the shrinkage of the reference series. One of the possible explanations is that the products of the pozzolanic reaction of glass contributed to a more intensive shrinkage of series, having up to 20% of glass replacing cement in the mentioned period. At 90 days, WG20 concrete has only 6.15% lower shrinkage than the reference concrete, while in concretes with a lower percentage of replacement of cement. this difference is even smaller. In accordance with the previously made statements, it can be concluded that in the period from day 90 to day 180, WG 35 concrete is the fastest in reaching the reference concrete value in terms of shrinkage.

### 3.9. Water Absorption

**Figure 10:**
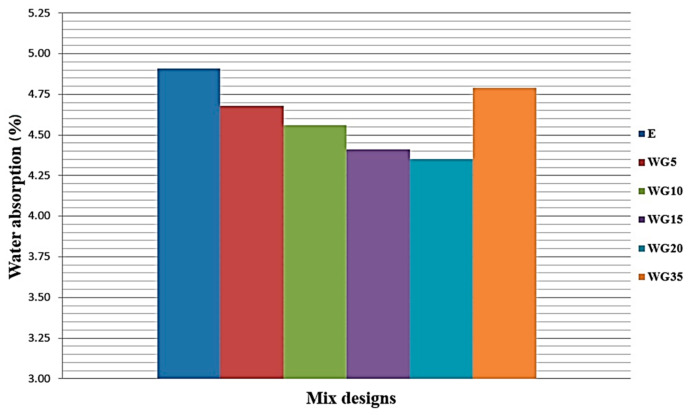
Impact of replacement of cement with CRT glass on water absorption of concrete under atmospheric pressure.

A graphical presentation of the impact of the replacement of cement with finely ground CRT glass in concrete on the water absorption is presented in Figure 10. It can be observed that with an increase in the percentage replacement of cement with CRT glass of up to 20%, the water absorption in relation to the reference concrete is reduced. Concretes WG15 and WG20 have 10.18% and 11.41% lower water absorption than the reference series. A reduction in water absorption can indicate a potential improvement in concrete durability. The reduction in water absorption can be one of the exact explanations for the increase in the compressive strengths of concrete up to the cement replacement level of 15%. The explanation for such an effect on the replacement of cement with glass can be sought in the creation of a more compact structure of concrete, which is contributed by the finely ground glass. The provided results are in accordance with the research in [24] and in [26]. The researchers in [24] concluded that the replacement of a part of cement with ground glass up to 15% results in a reduction in water absorption. Omran et al. [26] determined a reduction in water absorption in concretes, which were made using a ground food container glass as a replacement of a part of cement.

### 3.10. Testing Resistance to Sulfate Attack

**Figure 11:**
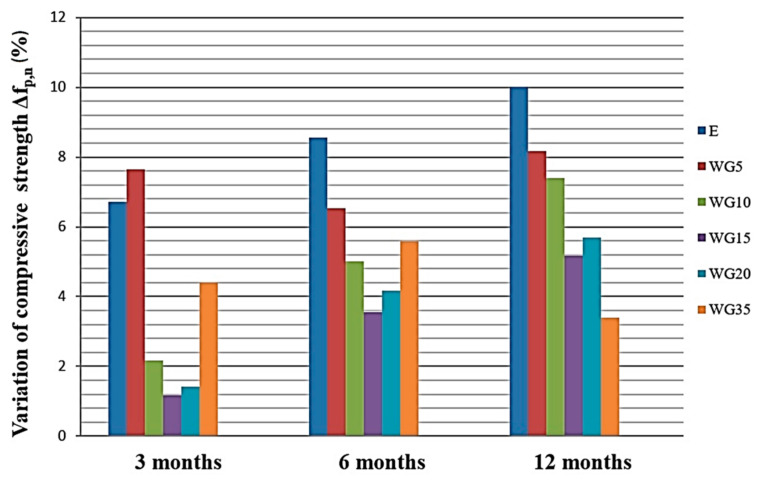
Variation of compressive strength of samples cured in solution of calcium-hydroxide (reference samples) and those cured in sodium-sulfate solution after 3, 6, and 12 months in %.

After the very impact of the presence of CRT glass on the compressive strength of concrete has been defined (3.2), one can observe the resistance to sulfate attack of the experimental concrete. Figure 11 shows the variation of the compressive strength (in percentage) of the concrete samples cured in a saturated solution of Ca(OH)_2_ (pH = 12.5) and of the samples cured in sodium-sulfate solution (pH = 7.5) at the ages of 3, 6 and 12 months. Not even after curing samples in a 5% solution of Na_2_SO_4_ for a year, has there been any variation of compressive strength higher than 10%, compared to the samples cured in calcium-hydroxide (reference samples). The highest reduction in compressive strength is determined by the reference concretes, which have no CRT glass added, and it is 10.01%. As can be seen in Figure 11, concretes where a part of cement is replaced with CRT glass have a lower decrease in compressive strength at the age of 1 year compared to the reference concrete. Moreover, it is important that the visual inspection of these samples does not reveal the occurrence of any damage in the form of cracks or scaling. Matos et al. [46] concluded that in the process of the pozzolanic reaction, glass uses up calcium-hydroxide, so it is not available for the reaction with sulfates. In this way, the emergence of gypsum is prevented. Researchers in [48,49] also came to the conclusion that finely ground glass in cement composites increases their resistance to sulfate attack.

### 3.11. SEM and EDS Analysis

**Figure 12:**
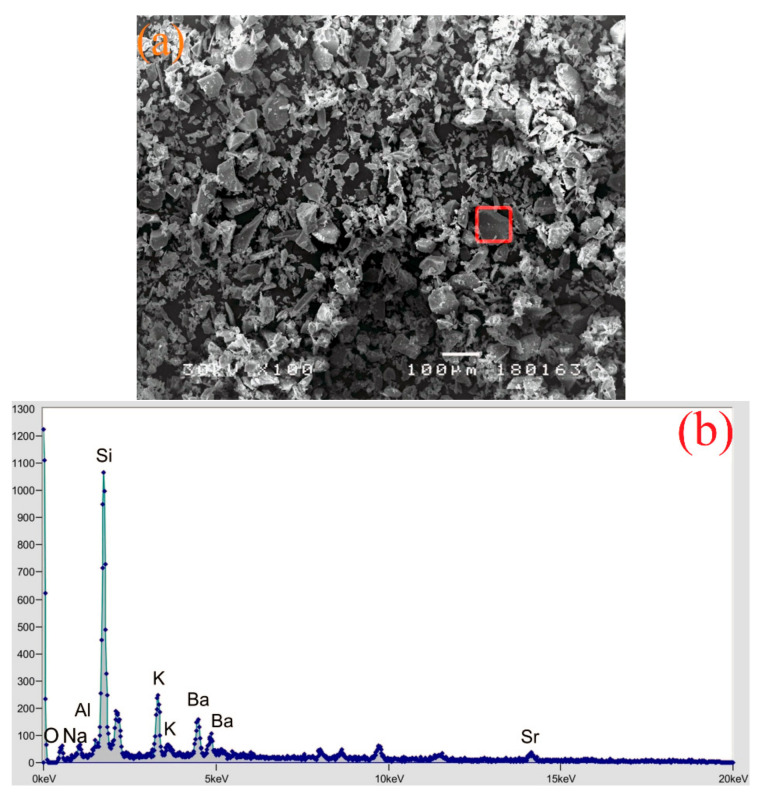
SEM analysis (**a**) and EDS spectroscopy of CRT glass (**b**).

**Figure 13:**
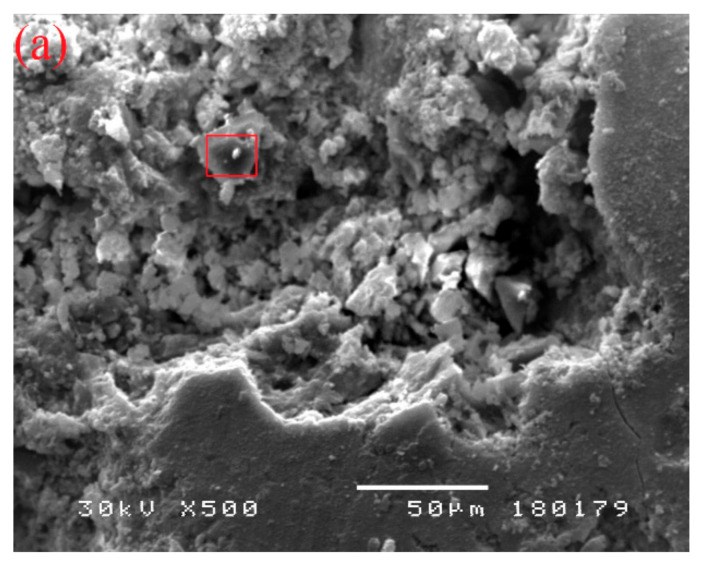

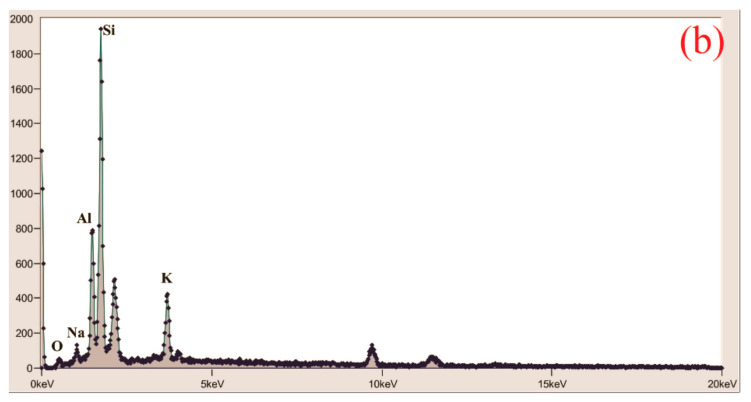
WG15 concrete, 500× magnification (**a**); EDS spectroscopy (**b**).

**Figure 14:**
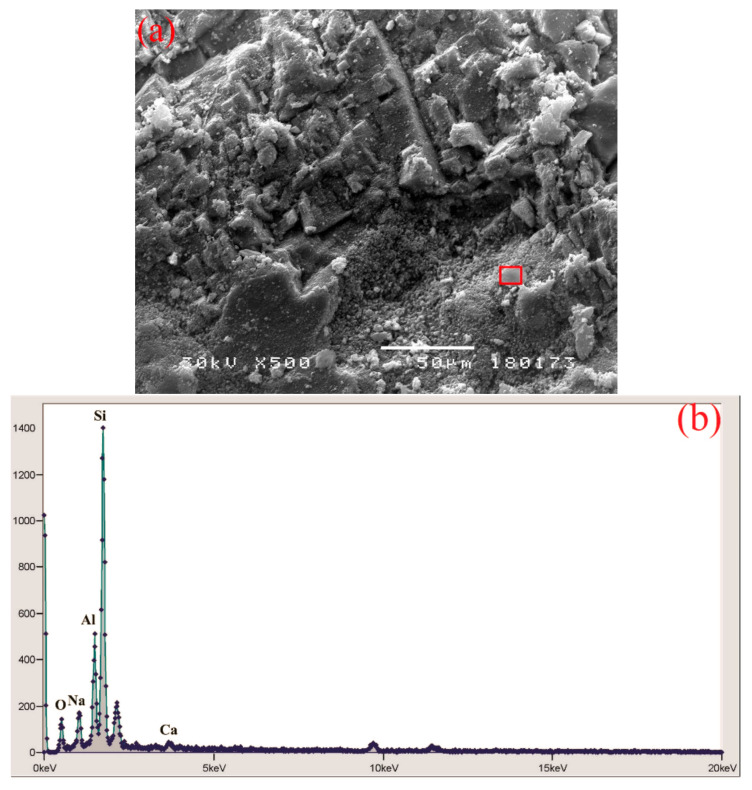
WG35 concrete 500 × magnification (**a**); EDS spectroscopy (**b**).

SEM and EDS analyses were conducted on the specimens of all experimental concrete series, while in the following text, only analyses referring to pure ground CRT glass, WG15, and WG35 are presented. Microscopic observation of finely ground CRT glass (Figure 12a) reveals that the grains have smooth surfaces and sharp edges. EDS spectroscopy (Figure 12b) determines the considerable presence of silicon. Considering that the panel glass was coated with protective layers during the service life of the electronic device, EDS spectroscopy reveals the presence of barium and strontium in traces, which originate exactly from the mentioned protective layers. On the basis of the EDS spectrum of WG15 concrete (Figure 13), it can be concluded that it represents a CRT glass grain enveloped in hydrate. The grain is firmly bonded with the cement matrix. A higher presence of sodium and potassium, in particular, is determined, which largely coincides with the EDS spectrum of pure glass. The increased presence of sodium can indicate that the EDS spectrum of the specimen of WG35 concrete (Figure 14) envelops the CRT glass grain. Since calcium presence is negligible, it is not a C-S-H phase. On the other hand, if the silicon peak size is observed, it corresponds to the concentration of silicon in CRT glass (Figure 12b), while in the case of aggregate grain, the presence of silicon is much more pronounced. The observed grain of CRT glass appears free, without a good bond with the cement matrix. This could serve as an explanation for the considerably lower mechanical strengths of this concrete series in relation to other concretes where cement was replaced by CRT glass. On the other hand, finely ground glass grains can play the role of a sealant, which results in reduced penetration of water through such concretes.

On the observed concrete samples, SEM and EDS analyses do not indicate the presence of an undesirable alkali-silicate reaction, more precisely the A-S-R gel, which is in agreement with the results of the experimental tests of this phenomenon.

## 4. Conclusions

Replacement of a part of cement with finely ground CRT glass in the amount of 5% has a negligible impact on the compressive strength of concrete, while replacing cement with 10% and 15% of ground glass has a positive effect on the mechanical strength of concrete, as it increases it. The replacement of cement in the quantity of 20% has practically no impact on the variation of compressive strength in comparison to the reference concrete, while the replacement in the amount of 35% has a considerable effect on compressive strength by reducing it in comparison to the reference concrete (around −25%).

The durability of concrete, in terms of the properties which were tested and whose results were presented in the paper, corresponds to the durability of the reference concrete up to the quantity of 15% of CRT glass replacing the cement. The concretes in which cement was replaced with glass in the amount of 20% reached the ultimate values in terms of resistance to freeze-thaw action and simultaneous action of freezing and defrosting salt. This makes their use problematic and questionable in the conditions where they undergo such influences, and their use cannot be recommended. Finally, concrete mixtures with the highest replacement of cement with CRT glass in the amount of 35% did not exhibit satisfactory durability.

In general, based on the presented results of experimental research, it can be concluded that concrete composition with replacement of a part of cement with finely ground CRT glass up to the amount of 15% meets the requirements for practical use of concrete which is exposed to the aggressive impacts of the environment.

A very important reason for using ground CRT glass as a cementitious material in practice is environment protection and solving the hazardous waste disposal matter. The amount of CRT glass that is still present and which is being collected by the recycling centers worldwide is remarkable and cannot be neglected. On the other hand, the production of cement concrete as the most used material in civil engineering is more than adequate to use up the entire available CRT glass as a replacement of a part of cement. Of course, it requires a certain investment, for instance, in the CRT glass mills, transport, storage, but its contribution to the protection of the environment is far more important.

## Figures and Tables

**Table 1:** Chemical composition of cathode ray tube (CRT) glass and cement.

	Chemical Compound									Physical Characteristics	
Binder	SiO_2_ (%)	Al_2_O_3_ (%)	Fe_2_O_3_ (%)	CaO (%)	MgO (%)	K_2_O (%)	Na_2_O (%)	SO_3_ (%)	LOI (%)	Specific Surface (Blaine) m^2^/kg	Density kg/m^3^
CRT glass	60.61	2.88	0.58	1.31	0.53	6.45	7.61	0.09	1.04	245	2840
Cement	19.30	4.28	2.87	62.8	2.22	0.91	0.21	3.05	2.26	394	3150

**Table 2:** Activity index of CRT glass.

Mortar Series	Activity Index (%)	
	28 Days	90 Days
*Criterion according to EN 450-1*	***min. 75%***	***min. 85%***
WG5	95	96
WG10	92	89
WG15	90	85
WG20	88	82
WG25	83	81
WG35	74	71

**Table 3:** Composition of experimental concrete mixtures.

Concrete	Aggregate			Cement	CRT	Water		Additive
	0/4 mm	4/8 mm	8/16 mm	CEM I 52.5R	<0.063 mm	City Wat. Supply	w/b	Super-Plasticizer
	kg/m^3^	kg/m^3^	kg/m^3^	kg/m^3^	kg/m^3^	kg/m^3^	-	kg/m^3^
E	774	414	612	400	-	175.3	0.438	2.40
WG5	774	414	612	380	20	175.3	0.438	2.40
WG10	774	414	612	360	40	175.3	0.438	2.40
WG15	774	414	612	340	60	175.3	0.438	2.40
WG20	774	414	612	320	80	175.3	0.438	2.40
WG35	774	414	612	260	140	175.3	0.438	2.40

**Table 4:** List of tests conducted on hardened concrete and employed standards.

Test Description	Specification
Compressive strength	EN 12390-3 [36]
Alkali-silicate reactivity	ASTM C227 [37]
Freeze-thaw resistance	SRPS U.M1.206 annex R [38]
Freeze-thaw resistance with de-icing salts-scaling	CEN-TS_12390-9 [39]
Depth of penetration of water under pressure	EN 12390-8 [40]
Resistance to wear by abrasion (Böhme test)	EN 1340:2012, annex H [41]
Tests of shrinkage due to drying in the air	UNI 6555 [42]
Water absorption	EN 13755 [43]
Testing resistance to sulfate attack	Own testing program

**Table 5:** Test results of resistance of concrete to simultaneous action of freezing/thawing and de-icing salt.

Concrete Series	Cumulative Amount of Scaled Material for Each Sample after 28 Cycles S_28_ [kg/m^2^]	Damage Description
E	-	N/A
WG5	-	N/A
WG10	-	N/A
WG15	-	N/A
WG20	0.02; 0.01; 0.09	Finer mortar damage
WG35	0.34; 0.33; 0.27	Surface damage, visible individual aggregate grains

## Data Availability

Publicly available datasets were analyzed in this study. This data can be found here: [https://nardus.mpn.gov.rs/handle/123456789/18135?fbclid=IwAR3VMPCrUU8da8atlDzwnMuxbpiL5zX2iAxg2I7VK-Fj-ZpeuIMe1GADN14] (accessed on 1 August 2021).
